# The Impact of *KIR* Polymorphism on the Risk of Developing Cancer: Not as Strong as Imagined?

**DOI:** 10.3389/fgene.2016.00121

**Published:** 2016-06-28

**Authors:** Danillo G. Augusto

**Affiliations:** Laboratório de Genética Molecular Humana, Departamento de Genética, Universidade Federal do ParanáCuritiba, Brazil

**Keywords:** killer cell immunoglobulin-like receptors, HLA genes, cancer, association, susceptibility

## Abstract

The polymorphism of killer cell immunoglobulin-like receptors (KIR) has been associated with several diseases, including infection, autoimmunity and cancer. KIR molecules are a family of receptors expressed on the surface of natural killer cells (NK), frontline defense of innate immunity against microorganisms and neoplastic cells. Some studies have shown conflicting results concerning the role that *KIR* polymorphism plays in tumor susceptibility, particularly in leukemia and lymphoma. Interestingly, the presence of HLA ligands is sometimes strongly associated with several types of cancer and apparently is not related with their interaction with KIR. This manuscript briefly reviews the uncommon polymorphism of *KIR* and critically summarizes the recent findings with regards of the importance of *KIR* variation for cancer susceptibility.

## Introduction

Natural killer (NK) cells were initially discovered because of their ability to kill virus-induced murine leukemic cells without prior sensibilization (Kiessling et al., 1975; Herberman and Ortaldo, 1981) and have been implicated in tumor surveillance and early recognition of microbial infections. NK cells present a variety of surface receptors that can either enhance or diminish their response against the target cell. That includes the killer cell immunoglobulin-like receptors (KIR), encoded by a region at chromosome 19 called leukocyte receptor complex (LRC; Wilson et al., 2000).

The *KIR* gene complex, located at 19q13.42 (Wende et al., 1999; Liu et al., 2000), consists of a cluster of homologous genes that has suffered extensive expansion and contraction (Wende et al., 1999; Martin et al., 2003). Fourteen *KIR* genes (*KIR2DL1-5, KIR2DS1-5, KIR3DL1-3, KIR3DS1*, and two pseudogenes (*KIR2DP1* and *KIR3DP1*) have been described. Not all *KIR* genes are present in all individuals; this uncommon presence/absence polymorphism generates a broad variety of haplotypes that differ among individuals and populations. The diversity of haplotypes combined with increased number of alleles in each locus make nearly impossible two non-related individuals to carry the same *KIR* variants.

The *KIR* polymorphism has been studied in several populations across the globe (Norman et al., 2001; Augusto et al., 2012b, 2013, 2015a, 2016; Hollenbach et al., 2012, 2013) and more than 500 *KIR* gene-content genotypes have been described among over 200 worldwide populations (Gonzalez-Galarza et al., 2015). However, allelic diversity is still poorly known.

The nomenclature of *KIR* genes is based on the structure of the mature protein. *KIR* genes encode two or three (2D or 3D) extracellular immunoglobulin domains that may have short (S) or long (L) cytoplasmic tails (Colonna et al., 1996). Except by KIR2DL4 (Kikuchi-Maki et al., 2003), all molecules that present long cytoplasmic tails are inhibitory and all KIR that present short tails transduce activating signals. *KIR* haplotypes can be divided in two major groups: (1) haplogroup A, which classically consists of a fixed number of genes, mostly inhibitory; and (2) haplogroup B, composed by a large variation of gene-content combinations, characterized by the presence of more activating genes (Uhrberg et al., 2002).

HLA (human leukocyte antigens) class I are MHC (major histocompatibility complex) molecules that bind self and non-self peptides and display them on the cell surface for recognition by appropriate cells of the immune system. Additionally, HLA are known ligands for KIR. HLA-C2 allotypes are recognized by KIR2DL1 (Wagtmann et al., 1995; Fan et al., 1996; Winter and Long, 1997); KIR2DS1 also binds HLA-C2, but at lower affinity (Stewart et al., 2005). HLA-C1 and some C2 allotypes are bound by KIR2DL2/3 (Winter et al., 1998), and predicted to be ligand for KIR2DS2/3. HLA function is primarily related to presentation of antigens and regulation of immune responses. During the course of evolution, HLA-A and HLA-B apparently kept their main role as T cell receptor (TCR) ligands while HLA-C seems to have had evolved as primarily KIR ligands (Older Aguilar et al., 2010). Still, several HLA-A and -B molecules interact with KIR. HLA-Bw4, that comprises about 40% of all HLA-B molecules (Müller et al., 1989) plus a subset of HLA-A (A^*^23, A^*^24, and A^*^32; Kostyu et al., 1980), are recognized by KIR3DL1 (Cella et al., 1994; Stern et al., 2008). Despite the lack of direct evidence (Gillespie et al., 2007; O'Connor et al., 2007), the homology with KIR3DL1 and the numerous disease association studies suggest that KIR3DS1 also recognizes HLA-Bw4. KIR3DL2 recognizes HLA-A3/A11 (Döhring et al., 1996; Hansasuta et al., 2004), B27 (Shaw et al., 2014; Hatano et al., 2015) and HLA-F (Goodridge et al., 2013). As product of gene conversion with *KIR3DL2*, KIR2DS4 also binds HLA-A11 (Graef et al., 2009) and HLA-F (Goodridge et al., 2013). HLA-A11 is also a ligand for KIR2DS2 (Liu et al., 2014).

*KIR* polymorphism has been associated with infection, autoimmunity and cancer (van der Slik et al., 2003; Khakoo and Carrington, 2006; Yamada et al., 2007; Kulkarni et al., 2008; Augusto et al., 2012a, 2015b). The importance of *KIR* for reproduction is also well-documented (Hiby et al., 2004, 2014; Trowsdale and Moffett, 2008; Nakimuli et al., 2015). There is strong evidence that *KIR* and *HLA* are coevolving as an integrated system (Augusto and Petzl-Erler, 2015) and that *KIR*-driven pressures are balancing *HLA* haplotypes (Capittini et al., 2012; Fasano et al., 2014; Nemat-Gorgani et al., 2014; Augusto et al., 2015a).

As consequence of infection or malignancy, abnormal cells may exhibit reduced expression of self-MHC molecules. NK cells are able to recognize and to attack those cells with low expression of self-MHC molecules (Parham, 2004). Over the last two decades, *KIR* genes have been reported among those most strongly associated with disease susceptibility. Because the recognition of HLA by KIR modulates NK function, and also because these cells are important for attacking tumors, variation in *KIR* and *HLA* have been thought to intensely interfere in the risk of developing cancer. This hypothesis was corroborated by case control studies that showed association of *KIR* presence/absence and leukemia (Verheyden et al., 2004; Zhang et al., 2010). However, as we critically discuss in this review, as more case-control studies have further been performed, it now seems that the role of *KIR* presence/absence variation in cancer susceptibility may not be as strong as initially believed.

## *KIR* polymorphism in leukemia

Acute lymphoblastic leukemia (ALL) is a malignancy in the bone marrow that leads to abnormal production and consequent excess of juvenile lymphocytes. ALL comprises ~75% of all cases of leukemia and normally occurs in children. ALL is a heterogeneous group of cancers that typically implicates B- or T-cell precursors, therefore subdivided in B-ALL or T-ALL. Differently, chronic lymphocytic leukemia (CLL) is a slow-growing tumor of lymphoid cells and usually occurs in individuals over 55 years of age. Myeloid leukemia causes rapid growth of myeloid cells; its acute form (AML) occurs either in children or in adults and its chronic form (CML) affects primarily adults.

*KIR* presence/absence in leukemia was initially explored by Verheyden et al., who reported *KIR2DL2* and *KIR2DS2* increased in patients (Verheyden et al., 2004). Both genes are present in haplogroup B; therefore, the authors could demonstrate that haplotype A was protective (Pc = 0.01). Primarily inhibitory genes compose haplotype A, suggesting that inhibitory *KIR* could protect against leukemia. Limitations of this study were the mixture of all four types of leukemia listed above within the patient group and the fact that the impact of *KIR* polymorphism in each form is not necessarily the same. Despite these limitations, the association of *KIR* polymorphism and leukemia appeared substantial. However, these results were not totally supported in a larger Chinese cohort (Zhang et al., 2010). Zhang and colleagues showed that *KIR2DL2* was not significantly increased in patients (*p* = 0.10). The presence of *KIR2DS4*, however, was significantly increased in the total patients' sample (OR = 1.76, *p* = 0.008), but this effect seemed to be driven by CML subgroup (OR = 3.29, *p* < 0.001). Although Zhang's study did not analyze *KIR* haplotypes, activating *KIR* were slightly more frequent in patients (not significant), what partially corroborated Verheyden's findings. In opposition to all these previous results, however, Middleton et al. showed that *KIR2DL2* was protecting against leukemia (Middleton et al., 2009).

In 2011, Almalte and colleagues analyzed a Canadian-French cohort composed by 145 B-ALL patients and 30 T-ALL and compared them to 245 controls (Almalte et al., 2011). In that study, the authors showed strong protective associations for the presence of all six activating *KIR* analyzed. They have not analyzed presence/absence of inhibitory genes, what challenge the interpretation those results due to the impossibility of analyzing the linkage disequilibrium between loci or verifying the *KIR* genotypes/haplotypes. Remarkably, an European cohort was further analyzed by Babor et al. and their results diverged from all former studies (Babor et al., 2012). Babor et al. reported no association between *KIR* presence/absence and leukemia, despite the fact that *KIR* frequencies in Babor's Canadian-French cohort did not differ from the cohorts from other studies. After that, another research group analyzed over 300 patients and performed another study (Oevermann et al., 2015). Applying careful and rigorous analyzes, Oevermann et al. corroborated Babor's results and reported absence of association of *KIR* presence/absence and leukemia. Lack of association was reported again in Thai patients (Vejbaesya et al., 2014).

Subsequent results brought some light to this discussion by showing the presence of homozygosity for haplotype A was associated with increased risk of developing leukemia in Hispanic, but not in Euro-descendants (de Smith et al., 2014). de Smith's explanation was that possibly the role played by *KIR* in leukemia may vary among ethnic groups.

Interestingly, three studies have shown stronger associations of leukemia with HLA: HLA-C2 (Babor et al., 2014) and specially HLA-Bw4 (Bw4/Bw4; OR = 3.9, *p* = 0.01; de Smith et al., 2014) and Bw4Ile80 (OR = 3.32, *p* = 0.0005). Although Bw4 and C2 are putative KIR ligands, due to conflicting results regarding *KIR* in leukemia, it is difficult to believe that these HLA associations are related to their interaction with KIR, but probably other HLA-related immune mechanisms.

Together, all these studies lead us to interpret that *KIR* genes probably don't play a major role in leukemia susceptibility, and this effect may vary in different ethnic groups. Additionally, *HLA* polymorphism has a stronger effect in leukemia susceptibility than *KIR*. This conclusion is also supported by another study, which showed only a trend of association for the presence of five or six activating *KIR* genes (*p* = 0.06), but a strong association for the presence of Bw4 (OR = 0.56; *p* = 0.005) in CLL patients (Karabon et al., 2011). Another interesting result from this same study is that, in general, the combinations *KIR3DL1*/*S1*+Bw4 presented similar odds ratios when compared to Bw4 alone. The association of the pair *KIR3DS1*+Bw4 (OR = 0.46; *p* = 0.003) being similar to Bw4 individually suggests that the effect appears to be driven mostly by Bw4. To explore *KIR*-*HLA* in the allelic level or expression studies like the one performed by Obama et al. (2007) could be a key to bring some light to this subject.

## Lymphoma and multiple myeloma

The presence of large tumor cells derived from a germinal center B cell, known as Hodgkin and Reed-Sternberg, characterizes Hodgkin lymphoma (HL; Re et al., 2005). Epstein-Barr virus (EBV) is the major environmental factor associated with HL; approximately 40% of HL patients in the Western community tested positive for EBV (Küppers, 2009). Considering the importance of *KIR* for virus elimination, it is plausible to consider them as candidate genes for HL association studies. A familial study with 90 French families and 255 first-degree siblings was the first analysis of *KIR* polymorphism in HL (Besson et al., 2007). They reported negative association for the presence of *KIR3S1, KIR2DL5, KIR2DS1* and *KIR2DS5* (0.42 < OR < 0.56; 0.006 < *P* < 0.05). In that same study, they could not replicate their own results in a case-control study with 68 patients. Lack of association was also reported in a Lebanese case control study with 41 patients and 120 controls (Hoteit et al., 2015). It is important to notice that both case-control studies that reported lack of association were composed of small samples, what makes difficult to exclude the relevance of *KIR* polymorphism for HL pathogenesis. Furthermore, a familial study is more powerful than a case-control study, especially in the example above, in which Besson et al. performed deep analyzes, including EBV status of each HL patient.

*KIR* variation was also studied in non-Hodgkin lymphomas and multiple myeloma. Similarly from what was shown for ALL, no associations were seen for individual *KIR* genes in diffuse large B-cell lymphoma (DLBCL; Vejbaesya et al., 2014). Despite the lack of association with *KIR* variation in DLBCL, significant association was reported for the presence of HLA-Bw4 (OR = 0.39; *p* = 0.003). Presence of HLA-Bw4 alone showed similar odds ratio when comparing to the receptor/ligand pair *KIR3DL1*+HLA-Bw4 (OR = 0.34; *p* = 0.0006), what suggests that *HLA* alone was driving the effect. In multiple myeloma, *KIR2DS5* and some alleles of *KIR2DS4* were associated with increased risk (Hoteit et al., 2014), but again, the sample size was not large enough to allow more conclusive assumptions. Comprehensive studies with large and well-characterized cohorts need to be performed to verify the real impact of *KIR*-*HLA* in these diseases.

## Breast cancer

A pilot study analyzed the presence/absence of *KIR* in breast cancer (Ozturk et al., 2012). In that study, the authors analyzed 33 patients and 77 controls and reported borderline associations: *KIR2DS1* associated with increased risk (*p* = 0.03) and *KIR2DL1* increased in controls (*p* = 0.02). In addition, the authors performed allelic typing for *KIR2DS4* and the alleles KIR2DS4^*^003/*4*/*6*/*7* were overrepresented in controls (*p* = 0.03). Although the authors suggested that *KIR* variation might be involved in breast cancer pathogenesis, the small cohort and the borderline associations didn't provide conclusive results. These suggestions could not be corroborated by another study in a larger cohort of predominantly euro-descendants from Brazil (230 patients and 278 controls; Jobim et al., 2013). Jobim et al. reported a strong association for the presence of *KIR2DL2* (OR = 2.7; *p* < 10^−8^) and for the presence of HLA-C1 (OR = 2.7; *p* < 10^−7^). The strong associations reported for breast cancer in the Brazilian cohort suggest that KIR2DL2 combined with its ligand C1 are, in fact, altering susceptibility to breast cancer. It is important to notice that the combination *KIR2DL2*+C1 in the absence of *KIR2DS2* presented odds ratio as strong as 9.9 (Pc < 0.001).

## Colon and rectal cancers

Absence of association of *KIR* with colorectal cancer was reported in Europeans (Middleton et al., 2007); different from Koreans, in which *KIR2DS5* was increased in patients (OR = 1.9; *p* = 0.0007; Kim et al., 2014). Al Omar et al. also reported lack of association of *KIR* and colon cancer (Al Omar et al., 2010); interestingly, they showed a strong association of the presence of HLA-Bw4 (Bw4Ile80, OR = 3.1, *p* = 0.0001; Bw4The80, OR = 0.3, *p* = 0.0001 in individuals KIR3DL1+Bw4+). HLA-Bw4Ile80 is a stronger ligand for KIR (Cella et al., 1994); although this association suggests that KIR may interfere in the colon cancer susceptibility, it is important to note the lack of association for KIR+HLA pairs.

## Other cancers

**Figure 1:**
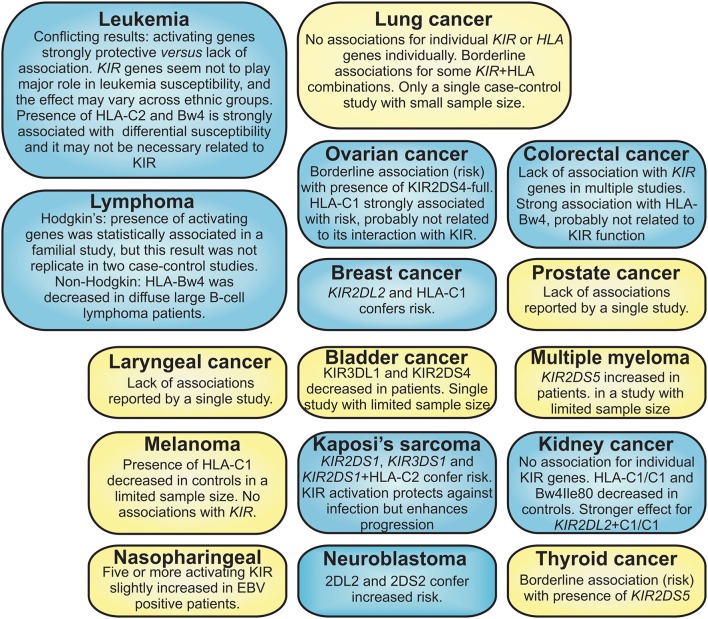
**Summary of the cancers for which ***KIR*** polymorphism have been analyzed**. Blue boxes = statistically significant association of cancer with *KIR* and/or HLA ligand; yellow boxes = borderline associations, lack of association or studies with reduced sample size.

Figure 1 and Table 1 summarize the associations and effect seen for *KIR* and ligands in several types of cancer. Nasopharyngeal cancer (NPC) is another example of neoplasm in which *HLA* polymorphism plays a major role in its susceptibility. In Chinese, HLA-B58 and HLA-A11 have been shown to confer risk and protection, respectively, for the development of NPC (Chan et al., 1983; Hildesheim et al., 2002; Lu et al., 2003). Even though HLA-A11 is a ligand for KIR3DL2 and KIR2DS4 (Döhring et al., 1996; Hansasuta et al., 2004; Graef et al., 2009), these relationships have not been investigated in NPC yet. The presence of five or more activating *KIR* conferred risk to EBV positive NPC patients; HLA-Cw4 was also reduced in NPC patients (Butsch Kovacic et al., 2005). *HLA* polymorphism seems to play a strong effect also in other cancers, such melanoma and ovarian, when comparing to a small or no effect of *KIR* for the susceptibility of those diseases (Naumova et al., 2005; Giebel et al., 2014).

**Table 1:** **List of the main association studies that analyzed ***KIR*** polymorphism in cancer**.

		**Ethnicity**	**OR/effect**	***P***	***N***	**References**
Leukemia	2DL2	Euro	Risk	0.007	94	Verheyden et al., 2004
	2DS2		Risk	0.022	94	
	A/A genotype		Protection	0.011	94	
	2DS4	Asian	1.76	0.008	263	Zhang et al., 2010
	2DS4 in CML		3.29	< 0.001	135	
	2DL2	Euro	0.61	0.029	158	Middleton et al., 2009
	2DL2 in CML		0.39	0.004	52	
	Bw4 Ile80		1.72	0.018	158	
	Bw4 Ile80 in AML		3.32	< 0.001	54	
	2DL2+C1		0.55	0.009	158	
	2DL2+C1 in CML		0.28	< 0.001	52	
	2DS2+C1		0.58	0.018	158	
	2DS2+C1 in CML		0.33	0.002	52	
	2DS1	Euro	0.55	0.020	100	Almalte et al., 2011
	2DS2		0.19	< 0.001	100	
	2DS3		0.32	< 0.001	100	
	2DS4		0.49	0.004	100	
	2DS5		0.32	< 0.001	100	
	3DS1		0.27	< 0.001	100	
	>4 Activating KIR		0.06	< 0.001	100	
	Lack of association with KIR	Euro	NA		220	Babor et al., 2012
	A/A	Hispanic	1.86	0.03	114	de Smith et al., 2014
	A/A	Euro	NA	0.37	76	
	Bw4/Bw4	Euro	3.93	0.01	76	
	Lack of association for KIR2DL1/S1 alleles	Euro	NA		320	Babor et al., 2014
	C1/C1 in ALL		0.69	0.005	320	
	Lack of association with KIR in B-CLL	Euro	NA		197	Karabon et al., 2011
	Bw4		0.56	0.005	197	
Lymphoma	3DS1 (familial study) in HL	Euro	0.44	0.006	345^*^	Besson et al., 2007
	2DL5 (familial study) in HL		0.56	0.02	345^*^	
	2DS1 (familial study) in HL		0.42	0.01	345^*^	
	2DS4full (familial study) HL		2.22	0.03	345^*^	
	Lack of association with KIR (case-control)		NA		68	
	Lack of association with KIR in HL	Arab	NA		41	Hoteit et al., 2015
	Lack of association with KIR in FL	Arab	NA		20	Khalaf et al., 2013
	Lack of association with KIR in DLBCL	Asian	NA		60	Vejbaesya et al., 2014
	Bw4 in DLBCL		0.39	0.003	60	
	3DL1+Bw4 in DLBCL		0.34	0.001	60	
Multiple myeloma	2DS4^*^001/002	Arab	Risk	0.04	34	Hoteit et al., 2014
	2DS5		Risk	0.007	34	
Nasopharyngeal	>5 Activating KIR	Asian	3.40	0.07	378	Butsch Kovacic et al., 2005
Breast cancer	2DL1	Euro	Risk	0.03	34	Ozturk et al., 2012
	2DS1		Risk	0.03	34	
	2DS4^*^003/4/6/7		Protection	0.03	34	
	2DL2	Euro	2.18	< 0.001	230	Jobim et al., 2013
	C1		2.71	< 0.001	230	
	2DL2+C1 in absence of 2DS2		9.95	< 0.001	230	
Colorectal cancer	Lack of associations	Euro	NA		128	Al Omar et al., 2010
	2DS5	Asian	1.9	0.007	241	Kim et al., 2014
	Lack of association with KIR	Euro	NA		75	Al Omar et al., 2010
	Bw4 Ile80 in 3DL1+Bw4+ individuals		3.10	< 0.001	75	
	Bw4 Thr80 in 3DL1+Bw4+ individuals		0.30	< 0.001	75	
	Lack of associations	Euro	NA		90	Middleton et al., 2007
Melanoma	Lack of association with KIR	Euro	NA		50	Naumova et al., 2005
	C2		0.27	0.017	50	
Ovarian cancer	Lack of association with KIR	Euro	NA		142	Giebel et al., 2014
	C1		3.07	0.002	103	
Kidney cancer	2DL3+C1	Euro	5.90	0.009	40	Al Omar et al., 2010
	2DL2+C1-		0.08	0.002	40	
Kaposi's sarcoma	2DS1	Euro	3.82	0.008	32	Guerini et al., 2012
	3DS1		4.00	0.006	32	
	2DS1+C2		4.24	0.01	32	
	KIR3DS1+Bw4 Ile80	Euro	0.60	0.01	250	Goedert et al., 2016
Lung cancer	2DL3+C1/C1	Euro	0.58	0.007	186	Al Omar et al., 2010
Prostate cancer	Lack of associations	Euro	NA		185	Portela et al., 2012
Bladder cancer	3DL1	Euro	Risk	0.011		Middleton et al., 2007
	2DS4		Risk	0.011		
Laryngeal cancer	Lack of associations	Euro	NA		70	Middleton et al., 2007
Thyroid cancer	2DS5	Arab	1.77	0.036	85	Ashouri et al., 2012
Neuroblastoma	2DL2	Euro	1.57	0.019	201	Keating et al., 2015
	2DS2		1.66	0.008	201	

Except for Besson et al. (2007), all cited manuscripts performed case-control studies. “Euro” includes European and Euro-descendant populations. OR = odds ratio; P = p-value; N = patient sample size; A/A = homozygote genotype for haplogroup A; CML = chronic myeloid leukemia; AML = acute myeloid leukemia; HL = Hodgkin's lymphoma; FL = follicular lymphoma; DLBCL = diffuse large B-cell lymphoma. Red = risk (OR > 1); blue = protection (OR < 1); green = not associated (NA);

* this is a familial study composed with 345 individuals in total. Not all families were informative for all analyzes.

The combination KIR2DL2+C1/C1 was strongly associated with protection in kidney patients (OR = 0.08; *p* = 0.002, *n* = 40 patients). Similarly from what was seen for breast cancer, the combination KIR-HLA showed stronger effect than either *KIR* or HLA isolated, suggesting the role of KIR-HLA combinations for the risk to develop this disease (Naumova et al., 2007; Al Omar et al., 2010; Giebel et al., 2014).

Activating *KIR* genes were associated with Kaposi's sarcoma (KS), a complication of KS-associated herpesvirus (KSHV) infection (Antman and Chang, 2000). Activating genes (*KIR2DS1, KIR3DS1*, and the combination *KIR2DS1*+HLA-C2) were significantly increased in individuals with classic KS (Guerini et al., 2012). Goedert et al. showed that KIR activation might decrease the risk of KSHV infection in an Italian cohort, while might enhance KSHV dissemination and progression to KS if infection occurs (Goedert et al., 2016).

## Concluding remarks

Despite the number of studies, it is still difficult to fully comprehend the role of *KIR* variation in cancer. One of the reasons is the reduced number of studies that analyzed large and well-characterized cohorts. Some studies have shown strong association with some types of cancer, but lack of association in several other studies and conflicting results suggest that the role of *KIR* presence/absence polymorphism may vary in different cancers. It is also clear that further studies with larger cohorts are needed.

Leukemia and lymphoma are examples of diseases for which mostly divergent results have been reported. It is interesting, however, that despite the conflict regarding *KIR*, the presence of HLA ligands has been consistently associated with different types of cancer. Even more interesting is the fact that many studies have shown that combinations *KIR*-*HLA* did not exhibit stronger effect than *HLA* alone. This suggests that the associations with *HLA* are possibly not related to KIR interaction. All these studies together suggest that *KIR* presence/absence polymorphism possibly does not play a major role in cancer. Considering the importance of NK for killing neoplastic cells, and the growing number of studies reporting *KIR*-*HLA* association with diseases, this conclusion can be quite intriguing.

It is important, however, to emphasize that lack of association with *KIR* presence/absence does not mean that *KIR* is not relevant for cancer. First, presence/absence polymorphism doesn't take the allelic polymorphism in consideration. *KIR* allelic variation is poorly known and rarely studied in diseases. Lack of genetic association does not discard the possibility of the cancer being associated with *KIR* differential expression levels, what confers another layer of complexity. Finally, the epigenetic mechanisms that regulate *KIR*-*HLA* should be studied especially in cancer, as it has been extensively demonstrated the importance of epigenetic regulation for tumor development. The comprehension of how *KIR*-*HLA* may be implicated in cancer is beyond presence/absence polymorphism, and perhaps beyond genetics. Different approaches have to be carefully considered, not only for *KIR*-*HLA*, but also for all genes that could impact cancer susceptibility.

## Author contributions

The author confirms being the sole contributor of this work and approved it for publication.

## Funding

I thank the Coordenação de Aperfeiçoamento de Pessoal de Nível Superior (Capes) and the Science without Borders Program for the research fellowship.

### Conflict of interest statement

The author declares that the research was conducted in the absence of any commercial or financial relationships that could be construed as a potential conflict of interest. The reviewer AC and handling editor declared their shared affiliation, and the handling editor states that the process nevertheless met the standards of a fair and objective review
